# Plasma tool as green route for incorporation of flame retardancy and ultraviolet protection of textile fabrics

**DOI:** 10.1038/s41598-026-47539-x

**Published:** 2026-04-16

**Authors:** Ahmed M. Abdel-Razik, Hanaa E. Nasr, Nour F. Attia

**Affiliations:** 1 Materials Testing and Surface Chemical Analysis Laboratory, Chemistry Division, National Institute for Standards, 136, 12211 Giza, Egypt; 2 Department of Polymers and Pigments, National Research Centre, Dokki, Giza, 12622 Egypt; 3 Gas Analysis and Fire Safety Laboratory, Chemistry Division, National Institute for Standards, 136, 12211 Giza, Egypt

**Keywords:** Plasma treatment, Surface treatments, Flame retardancy, Antibacterial properties, UV protection, Tensile strength, Chemistry, Materials science, Nanoscience and technology

## Abstract

Herein, gas plasma was used as an effective route for surface activation for easier incorporation of fire safety, bacterial growth inhibition and UV shielding functions for synthetic polyacrylic fabrics. Oxygen and nitrogen plasma were used with different time exposure for surface activation and their influence for activation was studied. Green nanocomposite-based coating layer was fabricated from ZnONPs prepared using molokhia stems extract of an average nanoparticle diameter of 6.2 nm in conjunction with sodium tripolyphosphate and tetra-n-butylammonium hexafluorophosphate as flame retardant agents. The dispersion of ZnONPs were dispersed in the nanocomposite using ultrasonication process. Afterwards, the plasma treated polyacrylic fabric was coated directly with the fabricated coating layer. The fire safety, antibacterial and UV shielding properties for the treated and untreated polyacrylic fabrics were investigated. The coated polyacrylic fabric achieved good flame retardant properties, recording a decrease in the rate of burning by 83% compared to blank polyacrylic. Additionally, the emission of toxic gases during combustion was suppressed compared to uncoated polyacrylic fabrics. The nanocomposite-based polyacrylic coating inhibits the growth of both gram-positive bacteria of *Staphylococcus aureus* and gram-negative bacteria of *Escherichia coli* achieving clear inhibition zone of 7.6 and 7.8 mm, respectively, compared to blank polyacrylic sample. The UV protection ability was enhanced significantly for coated fabric affording more than three-fold superior ultraviolet protection factor value than uncoated one. Moreover, the tensile strength was improved achieving reinforcement by ~ 10%.

**Supplementary Information:**

The online version contains supplementary material available at 10.1038/s41598-026-47539-x.

## Introduction

The textile materials are extensively used in various needs due to their excellent mechanical and flexible properties, this is in addition to their cost-effective price and abundance sources. However, due to their organic and synthetic precursors they suffer from various weakness such as high fire hazards, negative sensitivity toward UV rays and suitability for bacterial growth^1–3^. Hence, to address these drawbacks, special treatment should be conducted for fabrics before use for incorporation of flame retardancy, UV shielding and bacterial growth inhibition features^3–6^. The fabrics treatment mainly involves surface treatments via surface coating with a coating layer on textile surface through supramolecular interactions between coating layer and fabrics functional groups^7–9^. However, in case of synthetic fabrics such as polyacrylic fabric (PA), the poor suitable functional groups on PA surface for interaction with coating layer affords poor coating layer durability and performance. Thus, the surface modification and generating surface functional groups is a critical process for well coating process of PA fabric. On the other hand, the plasma is considered as clean and green tool for surface modification towards wettability, switching it from hydrophobic to hydrophilic surface^10,11^. Therefore, plasma treatment of fabrics is a vital step for inclusion of functional groups and radicals which is essential for chemical fabric treatment^12^. Importantly, the plasma treatment is essential for inclusion of textile surface with functional groups and in turn facilitating the coating with smart and green coating layer affording the fabrics with flame retardancy feature, UV shielding and antibacterial growth properties. Hence, in this study, green nanocoating layer composed from ZnONPs prepared using green extract in addition to fixed mass of tetra-n-butylammonium hexafluorophosphate (TBHP) and sodium tripolyphosphate (TPP) was fabricated. Then, the PA fabrics were treated with different plasma gases at different time exposure. Afterwards, the plasma treated PA fabrics were treated directly with the fabricated coating. The flammability hazards, UV protection, antibacterial and the tensile strength properties of the uncoated and coated PA were studied. Additionally, the surface morphology of the uncoated and coated PA fabrics was studied.

## Experimental

### Materials

Polyacrylic fabric (PA) was provided from resident market, Saudi Arabia. Molokhia stems were obtained from resident market, Egypt. Zinc acetate dihydrate and NaOH pellets were acquired from El Naser Pharmaceutical Chemicals, Cairo, Egypt. Sodium tripolyphosphate (TPP) and Tetra-n-butylammonium hexafluorophosphate (TBHP) were provided by Merck and Alfa Aesar, respectively. The deionized (DIW) was utilized for all processes.

### Preparation of ZnONPs based-nanocomposite coating layer

The molokhia stems extract was prepared based on our reported method^13^, however, the ZnONPs were prepared according to the reported method^14^. For ZnONPs preparation details, in a glass beaker contains 50 mL of DIW 2 g of Zinc acetate dihydrate was dissolved with magnetic stirring. Then, 20 mL of NaOH solution was added ( NaOH solution was made via dissolving 500 mg in 20 mL DIW) to previous solution. Then, 150 mL of molokhia stems extract was add to the ZnO solution followed by magnetic stirring for 3 h under heating (90 °C)^14^. After cooling to room temperature, 1 g of each TBHP and TPP was added separately and followed by magnetic stirring for1 h and followed by ultrasonication for 30 min as tabulated in Table 1.

### Plasma treatment and coating process of PA fabrics

The PA fabrics were treated with radiofrequency plasma using the Harrick plasma system. The plasma gas treatments were conducted under a radiofrequency power of 30 W for 60- and 90-min exposure, separately, using ultra-pure nitrogen and oxygen gases as plasma gas using 300 mtorr, individually. After the PA samples were treated with plasma gas using O_2_ and N_2_ at different times were immersed directly in the coating dispersion solution developed in Sect.  2.2 for 10 min and then squeezed, exposed to thermal curing for 5 min at 135 °C and finally dried in air. The last step was repeated three times. The sample denoted as PA-ZnONP refers to PA fabric coated with nanocoating layer without plasma treatment. However, the samples denoted as PA-N_2_-ZnONP-60(90) refers to PA fabric treated with N_2_ plasma gas coated with nanocoating layer and number refers to plasma treatment time in min. Also, the samples denoted as PA-O_2_-ZnONP-60(90) refers to PA fabric treated with O_2_ plasma gas coated with nanocoating layer and number refers to plasma treatment time in min (Table 1).

**Table 1:** Composition of plasma treated PA-nanocomposites.

Sample code	TPP (g)	TBHP (g)	Add on %
PA	0	0	0
PA- ZnONP	1	1	9
PA-N_2_-ZnONP-60	1	1	11
PA-N_2_-ZnONP-90	1	1	12
PA-O_2_-ZnONP-60	1	1	14
PA-O_2_-ZnONP-90	1	1	16.2

### Characterization

The surface microscopic images of PA and coated PA fabrics and their derived char were taken using a scanning electron microscope (SEM), located at Grand Egyptian Museum Conservation centre (GEM-CC). The dispersion and size of ZnONPs in the developed nanocomposite-based coating was scrutinized using Transmission Electron Microscope. The tensile strength and elongation properties of PA and coated PA were studied using H1-5KT/S model tensile testing machine based on the ASTM D 5035:1995^15^. The fire hazards feature of PA and coated PA fabrics were studied using FTT (UL94) horizontal test based on the standard test method ISO 3795^14,16–18^. Additionally, the flammability properties were further evaluated using limiting oxygen index (LOI) based ISO 4589-2^19^, moreover, the emission of toxic gases of NO, NOx, CO, CO_2_ and SO_2_ during LOI test was measured using testo gas analyser equipment. The UV protection factor for PA and treated PA was evaluated based on the AATCC test 183–2010 method^20^ utilizing JASCO (V-750) spectrophotometer. The bacterial growth inhibition for PA and treated PA was evaluated against *Staphylococcus aureus* and *Escherichia coli bacteria* using AATCC 147 method^21^. The mean clear antibacterial inhibition zone was determined following this equation W = (T-D)/2, T refers to sum of the width of the sample under test and clear antibacterial inhibition zone (mm). However, W refers to the width of the clear antibacterial inhibition zone only (mm) and D represents the width of the sample under test alone (mm)^21^.

## Results and discussion

### Synthesis and elucidation of developed nanocomposite-based coating layer on PA fabric

**Scheme 1:**
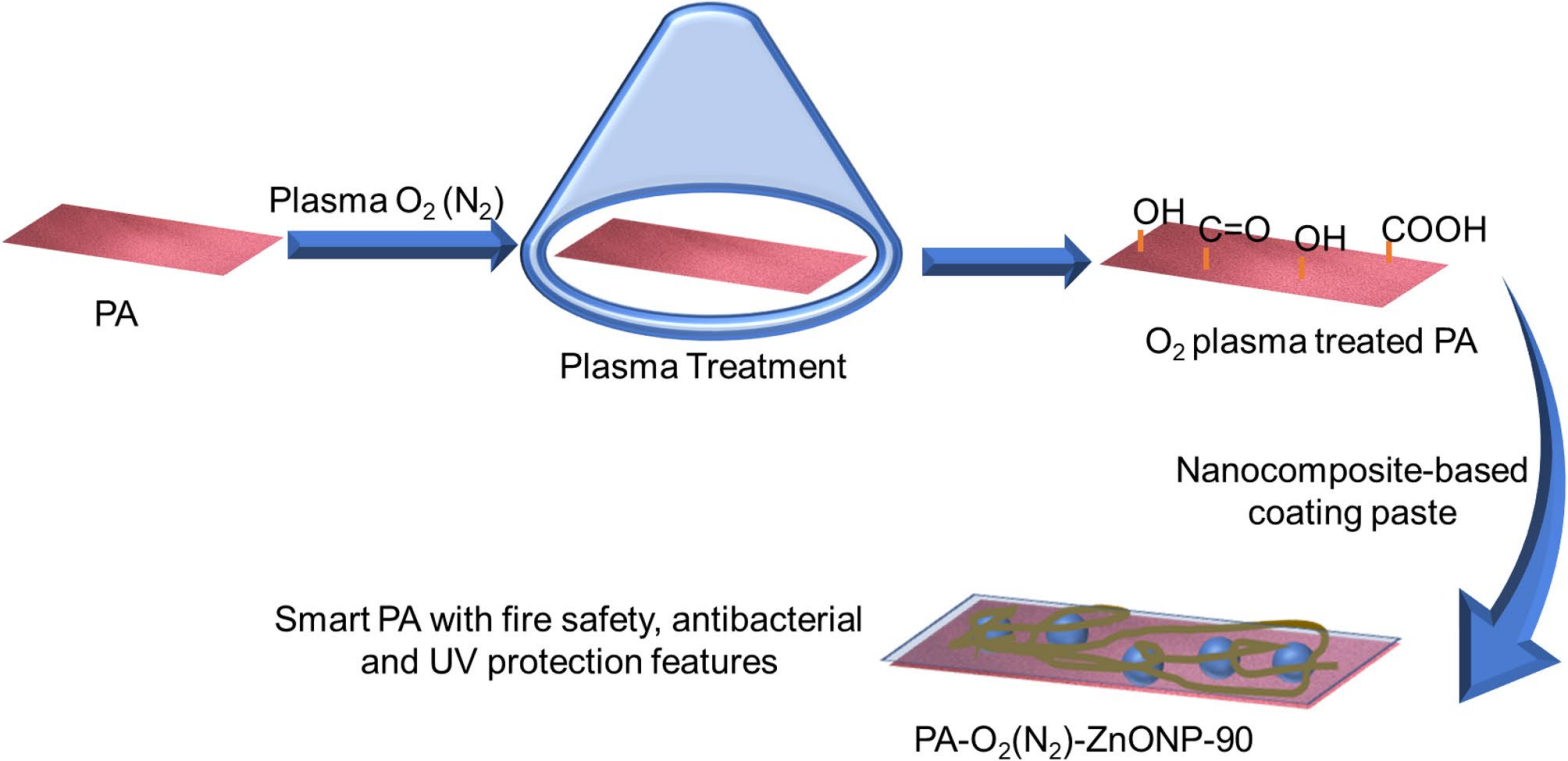
Schematic representation displaying the facile plasma activation of PA fabric surface and green coating of PA activated surface with multifunctional coating layer.

The plasma gas was used as an effective tool for the incorporation of functional groups on the surface of synthetic PA fabric for effective coating process by the fabricated nanocomposite-based coating layer for integrating new functions. Thus, two plasma gases of N_2_ and O_2_ gases were used and two exposure times of 60 and 90 min were used individually for each gas. Afterwards, the plasma treated PA fabric was coated with greenly synthesized nanocomposite-based coating fabricated from ZnONPs prepared using molokhia stems extract and TPP and TBHP with similar mass loading as displayed in Scheme 1. It is obviously noted that, the role of plasma treatment for incorporation of more mass loading of coating layer on PA surface (Table 1). Additionally, practical experiment was conducted for proving the inclusion of higher oxygenated functional groups in O_2_ plasma treated PA fabric. Therefore, water droplets were dropped on the surface of plasma untreated and O_2_ plasma treated PA fabrics, displaying the behavior. It is clearly observed the hydrophobicity of PA fabric which is plasma untreated and higher wettability for O_2_ plasma treated PA fabric as shown in Fig. S1. This corroborated the inclusion of oxygenated functional groups on PA surface which in turn responsible for the adhesion and interaction with the coating layer.

**Fig. 1:**
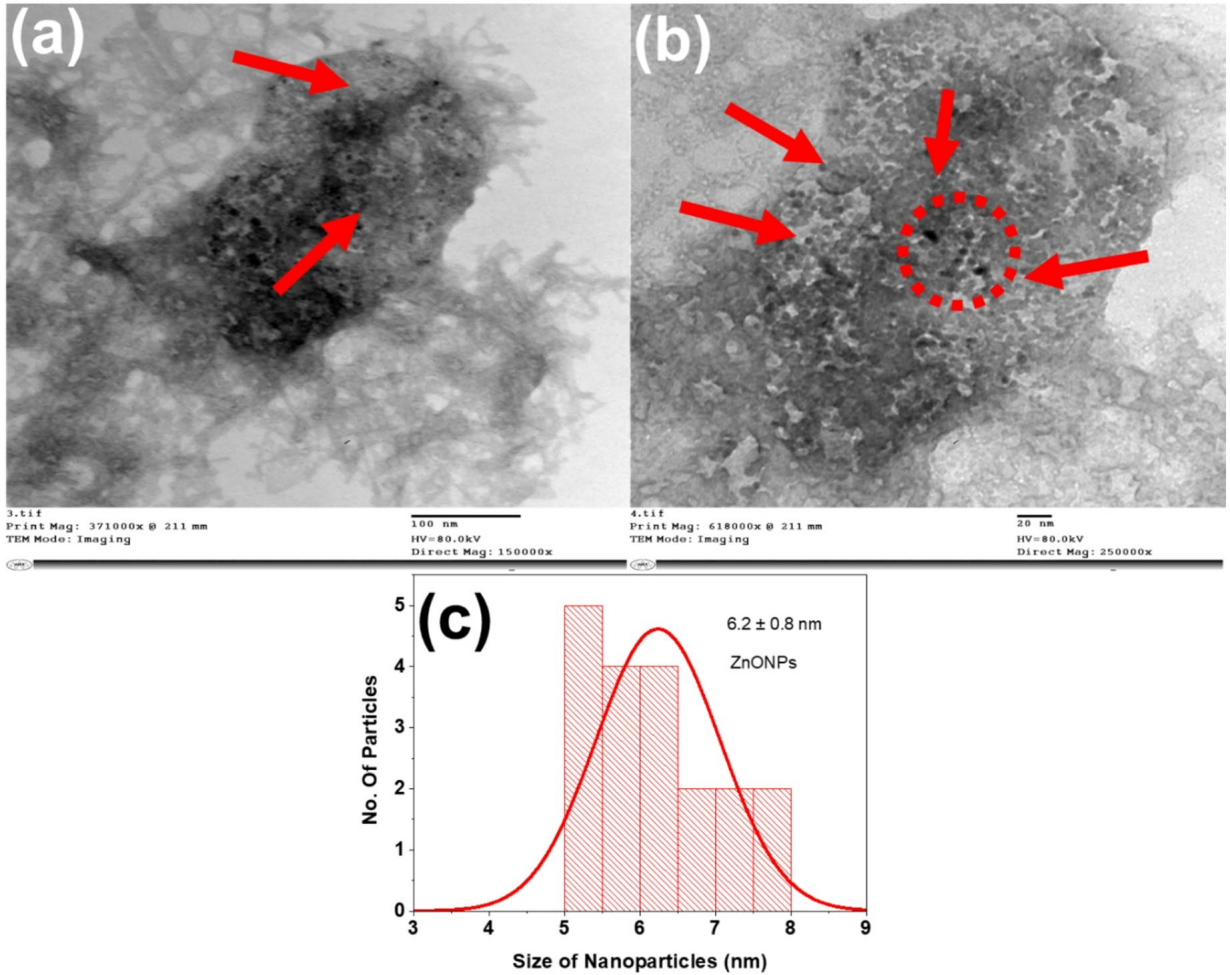
TEM images of (**a**) nanocomposite-based coating ZnONP-TPP-TBHP, (**b**) nanocomposite-based coating ZnONP-TPP-TBHP at high-magnification and (c) histogram of ZnO nanoparticle distribution.

Interestingly, the O_2_ plasma treatment has superior role compared to N_2_ plasma in improving the mass loading of coating layer on PA fabric as shown in Table 1. This indicates the oxygenated functional groups created are more efficient for promoting interaction with coating layer (Table 1). On the other hand, the dispersion of ZnONPs on the developed nanocomposite-based coating was studied using TEM. Thus, Fig. 1a represents the TEM image of nanocomposite-based coating composed from ZnONP-TPP-TBHP, which displayed the good dispersion of nanoscale ZnONPs in the nanocomposite-based coating. This is obviously visualized in high-magnification TEM image (Fig. 1b), and the mean diameter of the dispersed ZnONPs was determined to be 6.2 nm as shown in Fig. 1c. This affirms the successful synthesis of nanocomposite-based coating layer.

**Fig. 2:**
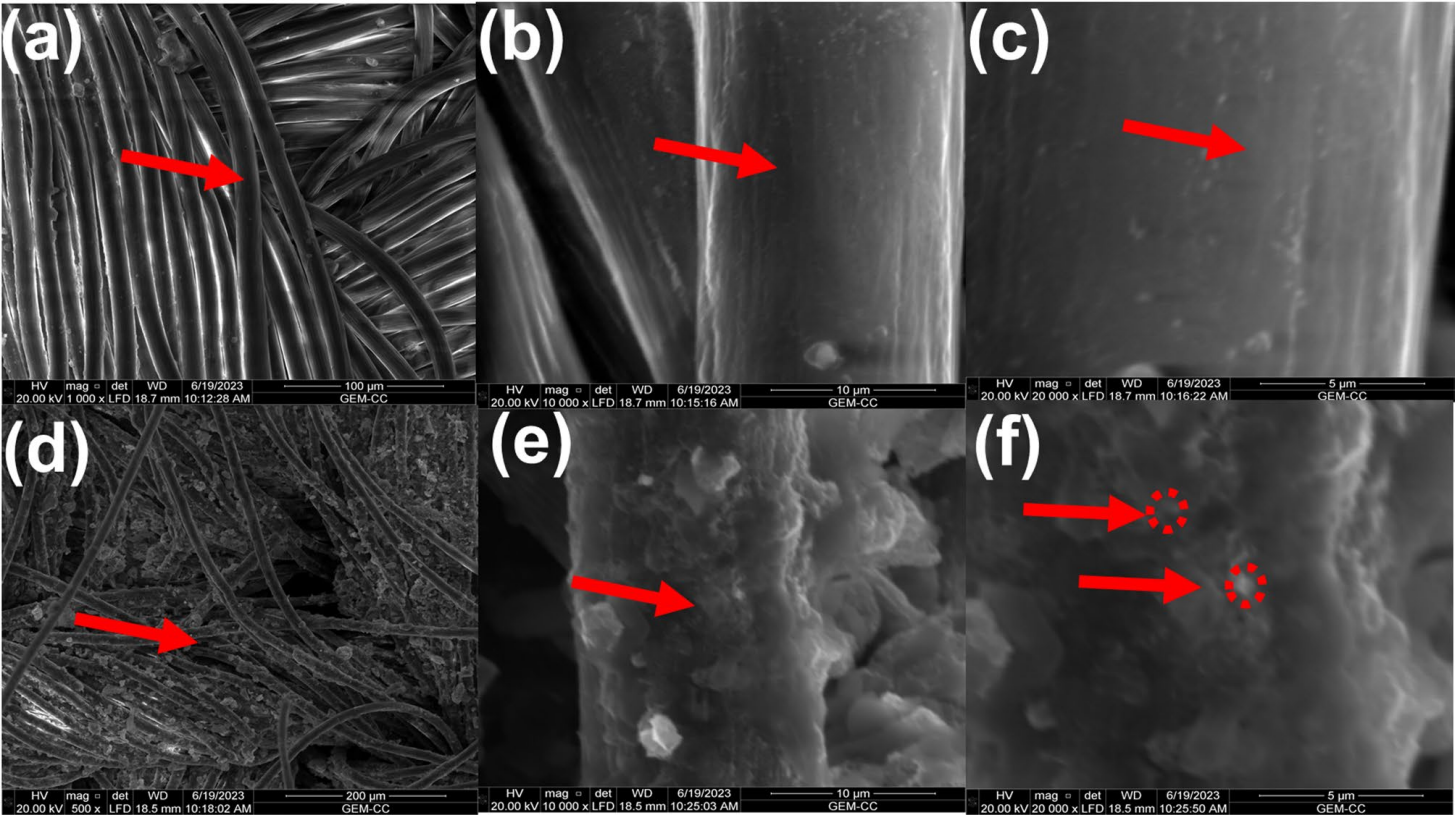
SEM images of (**a**) Polyacrylic fabric (PA), (**b**-**c**) PA fabric at high-magnification, (**d**) PA-N_2_-ZnONP-90 and (**e**-**f**) PA-N_2_-ZnONP-90 at high-magnification.

Interestingly, the surface morphology of the PA and coated PA fabric was examined using SEM. Thus, Fig. 2a shows the surface morphology of PA fabric, displaying the smooth surface of fibres. This was obviously seen at high-magnification SEM images (Fig. 2b-c). However, after coating the plasma treated PA fabric surface with nanocomposite-based coating layer fabricated from ZnONP-TPP-TBHP (sample: PA-N_2_-ZnONP-90), a rough surface of PA fibers was clearly noticed (Fig. 2d-e), this proves the existence of coating layer on the surface of PA fibers. This was further corroborated from high-magnification SEM image which visualizing the ZnONPs wrapped with molokhia stems extract compounds and TPP and TBHP layers on the surface of PA fibers as highlighted via red arrows (Fig. 2f). This provide additional evidence of the coating of PA surface with nanocomposite-based coating layer.

### Flammability properties of coated PA fabric

The fire hazards of PA and coated PA fabrics were investigated using rate of burning (RB) horizontal test using the standard method ISO 3795^14,16–18^ and limiting oxygen index (LOI) according to ISO 4589-2^19^. The RB of PA fabric was found to be very high recording RB of 745 mm/min (Table 2)^22^. This high flammability is due to its organic synthetic composition which easily ignited once exposed to fire source^22^. Also, the fire hazards of PA were elucidated by lower LOI value of 13.4% (Table 2). However, once the nanocomposite-based coating layer was coated on PA surface without plasma treatment, the fire risks of the coated PA (PA-ZnONP) was decreased recording RB of 336 mm/min (Table 2) achieving reduction by 55% and LOI value of 16%. This noted fire safety was attributed to the synergistic role of phosphate-based compounds (TPP and TBHP) that triggers PA fibers for forming char layer between flaming and burning zones offering retardation to heat and mass transfer^17,18,22,23^. Similar flame retardancy behaviour was noticed in PA-N_2_-ZnONP-60 sample recording 55% reduction in RB (Table 2). Also, this noticed fire safety was corroborated with higher LOI value compared to blank PA (Table 2).

**Table 2:** The fire hazards properties of PA and PA coated samples.

Sample Code	Rate of Burning (mm/min)	Reduction (%)	LOI (%)
PA	745	0	13.4 ± 0.152
PA- ZnONP	336	55	16 ± 0.152
PA-N_2_-ZnONP-60	336	55	16 ± 0.34
PA-N_2_-ZnONP-90	220	70	15.9 ± 0.057
PA-O_2_-ZnONP-60	152.7	80	16 ± 0.208
PA-O_2_-ZnONP-90	126	83	16.5 ± 0.17

**Fig. 3:**
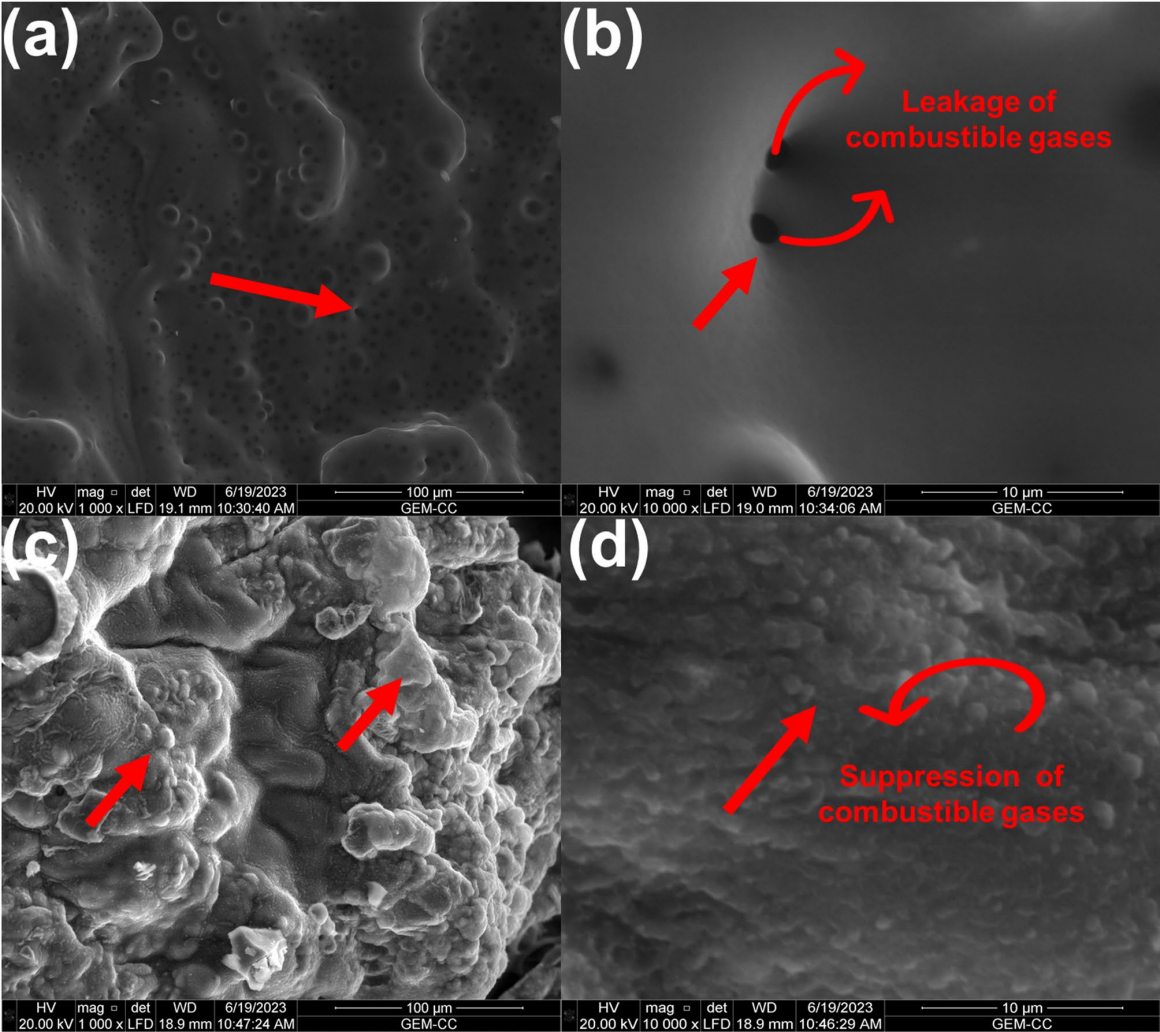
SEM images of char layer after UL94 test of (**a**) uncoated PA, (**b**) uncoated PA at high-magnification, (**c**) PA-N_2_-ZnONP-90 sample and (**d**) PA-N_2_-ZnONP-90 sample at high-magnification.

However, after 90 min exposure of plasma treatment, the add on % of nanocomposite-based coating increased and in turn displaying superior flame retardancy effect for PA-N_2_-ZnONP-90 compared to 60 min plasma time exposure (PA-N_2_-ZnONP-60) for the same gas plasma. Thus, the RB was reduced by 70% as indicated in Table 2. This flame retardancy action was corroborated with limiting oxygen index (LOI) value reaching ~ 16% compared to 13.4% for uncoated PA (Table 2), but almost similar value to PA-N_2_-ZnONP-60 (Table 2). Interestingly, once PA fabric treated with O_2_ plasma was coated with developed coating layer, the fire safety was significantly improved recording reduction in RB by 80 and 83% in PA-O_2_-ZnONP-60 and PA-O_2_-ZnONP-90, respectively. This superior flame retardancy effect was stemmed from the higher add on% (Table 1) of the nanocomposite-based coating layer on PA surface which in turn facilitates PA fibers for protective char layer formation. Moreover, the reduction in the fire hazards of O_2_ plasma treated samples was supported with higher LOI values recording 16.5% for PA-O_2_-ZnONP-90 sample. This elucidated the significant role of gas plasma treatment for incorporation of fire safety for PA. Moreover, the type of plasma gas played a significant role for improving the coating layer add on% (Table 1), hence, the O_2_ plasma treatment improves the interaction between the coating layer and PA surface and in turn affords better flame retardancy action. Interestingly, the char layer formed over the surface of the coated PA fabric suppressed the emission of toxic gases during the combustion (measured during LOI test), recording reduction in emission of CO_2_, NOx and NO gases by 16, 41 and 40.5% respectively, in PA-O_2_-ZnONP-90 sample compared to uncoated PA (Table S1). Additionally, records reduction in the emission of CO gas by ~ 5% and emission of SO_2_ gas (The sulfur originated from residue remained due to the usage of sulfonated based detergent during washing process) by ~ 17% compared to uncoated PA fabric (Table S1). The noticed flame retardancy action of plasma treated coated PA fabric was further elucidated using microscopic images of developed char layer for uncoated and coated PA. Thus, Fig. 3a visualize the char layer (derived after UL94 test) of the uncoated PA fabric displaying the existence of pores which are responsible for leakage of combustible gases and in turn feed the flaming zone. This is evidently seen in high-magnification SEM image which clearly indicating the pores (Fig. 3b). However, for PA sample coated with the nanocomposite-based coating layer in PA-N_2_-ZnONP-90 sample, condensed char layer was visualized as seen in Fig. 3c, this is due to the synergistic flame retardancy action occurred between the phosphate-based compounds in coating layer^17,18,23^. This compact and condensed char layer formed was clarified in high-magnification SEM image (Fig. 3d) displayed the role of protective layer as barrier hinder the heat and mass transfer and suppressed the combustible gases release. Although, good flame-retardant property was attained in this study via plasma treated coated samples compared to uncoated PA, however, it needs further study to strengthen the char layer to achieve zero rate of burning using UL94 test *(Horizontal test)* and higher LOI value (higher than 25%).

### Tensile strength, antibacterial and UV shielding properties of coated PA fabric

The impact of nanocomposite-based coating on the tensile strength (TS: which is represented via the maximum force at break by N) and elongation percent (EP) of PA were investigated using ASTM D 5035^15^ and data were tabulated in Table 3. The TS of PA was found to be 202 N associated EP of 19.7%^22^. However, upon coating of nanocomposite-based coating without plasma treatment for PA-ZnONP, the TS and EP were found to be 208.6 and 19.1% respectively. This enhancement in TS of PA-ZnONP compared to PA was attributed to the coating of nanocomposite on the surface of PA yarn reinforced its strength^23^. Adhering to the same reinforcement trend, TS of PA-N_2_-ZnONP-60 was improved recording 222.8 N in conjunction with EP of 17.7 (Table 3). This improvement in TS could be stem from the strengthening of supramolecular interactions between nanocomposite-based coating and plasma treated PA surface functions and in turn reinforced their fibers. However, upon high exposure of N_2_ plasma (90 min) for PA-N_2_-ZnONP-90, the TS was maintained almost similar to PA, in addition to inferior in EP (Table 3). This could be attributed to the weakness occurred in fiber due to plasma treatment for longer exposure. This phenomenon was noticed in PA-O_2_-ZnONP-60 (Table 3). However, for PA-O_2_-ZnONP-90, the TS and EP were found to be 217.7 N and 16.5 respectively, achieving reinforcement in coated PA. This reinforcement could be stemmed from (1) the strengthening occurred due to the decoration of ZnONPs based coating on the surface of PA fibers and (2) advancing in the supramolecular interactions between nanocomposite-based coating and plasma treated PA fabric.

**Table 3:** The tensile strength and elongation percent properties of PA and coated PA fabrics.

Sample code	TS (*N*)	Elongation (%)
PA	202 ± 20	19.7 ± 1.5
PA- ZnONP	208.6 ± 21	19.1 ± 3.3
PA-N_2_-ZnONP-60	222.8 ± 16	17.7 ± 3.3
PA-N_2_-ZnONP-90	199 ± 6.7	16.4 ± 0.7
PA-O_2_-ZnONP-60	196 ± 58	18.4 ± 3.1
PA-O_2_-ZnONP-90	217.7 ± 26	16.5 ± 1.2

**Fig. 4:**
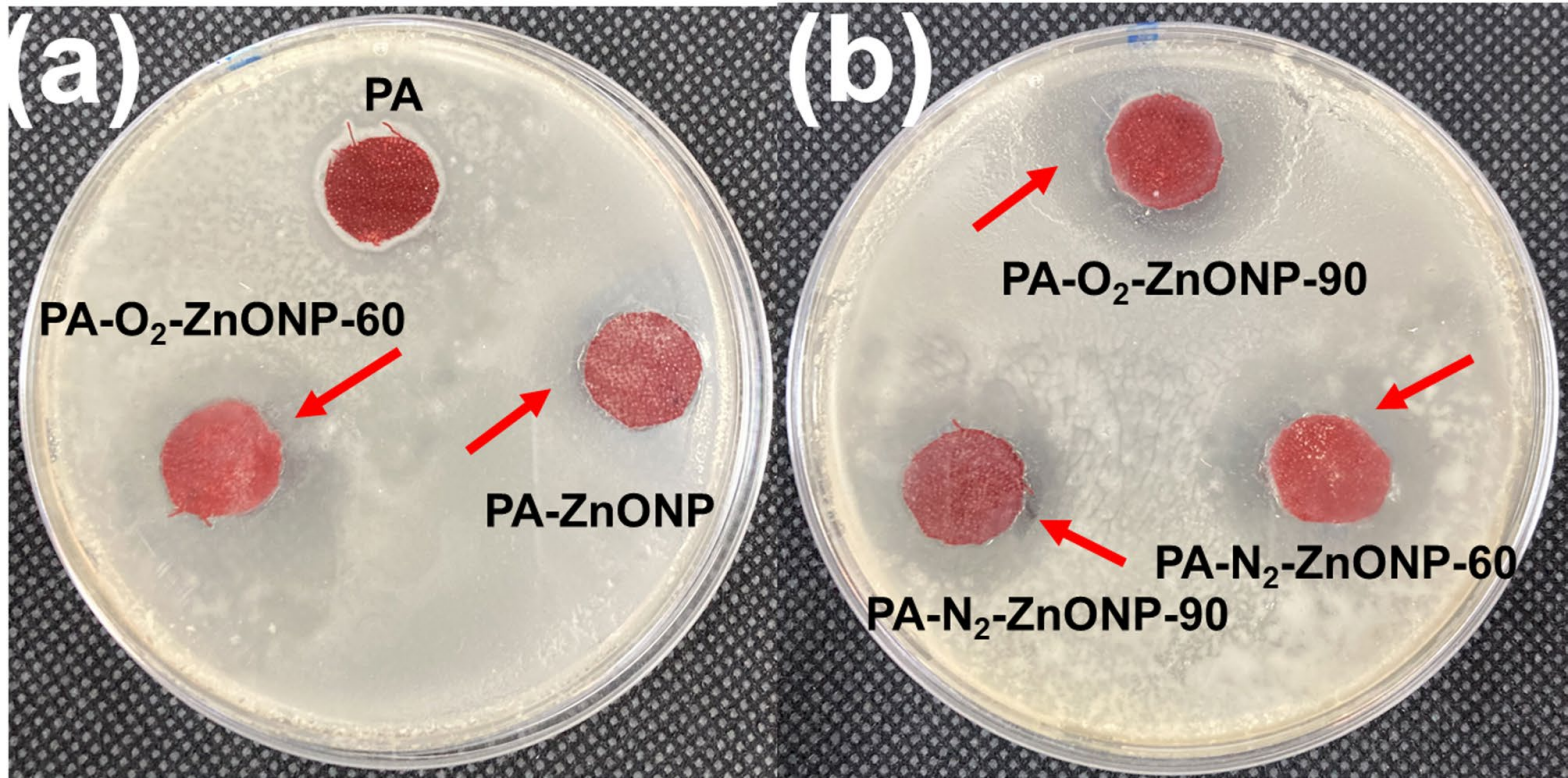
The digital photos of the clear antibacterial inhibition zone of PA fabric and coated PA fabric against S. *aureus* bacteria.

On the other hand, inclusion of antibacterial feature for fabrics is crucial process due to the suitability of fabrics for bacterial growth. Therefore, the ability of coated PA for inhibition of bacterial growth was evaluated based on standard method^21^. The antibacterial evaluation for uncoated and coated PA was conducted against both gram-positive and gram-negative bacteria of *Staphylococcus aureus (S. aureus)* and *Escherichia coli (E. coli)*, respectively, and the results were shown in Fig. 4, Fig. S2 and tabulated in Table 4. The uncoated PA has no action against both types of bacteria recording zero mm of clear antibacterial inhibition zone as shown in Fig. 4 Fig. S2 and Table 4. However, once nanocomposite-based coating layer was coated on PA without plasma treatment, positive antibacterial effect was noticed recording ~ 5 mm of clear inhibition zone for both bacterial strains (Fig. 4 and Fig. S2). Interestingly, the plasma treated coated PA fabrics achieved good antibacterial behaviour (Fig. 4) reaching clear antibacterial inhibition zone of 7.6 mm in PA-O_2_-ZnONP-90 sample against *S. aureus* bacteria (Fig. 4) and 7.8 mm in PA-N_2_-ZnONP-90 sample against *E. coli* bacteria as seen in Fig. S2 (Table 4). This positive antibacterial effect against *S. aureus* and *E. coli* bacteria could be attributed to the synergistic antibacterial effect stemmed from antibacterial capability of ZnONPs and phosphate-based compounds TPP and TBHP, in addition to molokhia stems extract compounds^14,18^. This antibacterial feature was enhanced upon plasma treatment, which afford good interaction and adhesion of convenient coating layer add on% (Table 1) on the PA surface and in turn, facilitates good inhibition to bacterial growth.

**Table 4:** Clear antibacterial inhibition zone for PA and coated PA fabrics against gram-positive (*Staphylococcus aureus)* and negative-bacteria (*Escherichia coli)*.

Sample Code	Clear inhibition zone (mm) Staphylococcus aureus	Clear inhibition zone (mm) Escherichia coli
PA	0	0
PA- ZnONP	4.66 ± 1.2	5 ± 1.3
PA-N_2_-ZnONP-60	6.33 ± 1	7 ± 0.5
PA-N_2_-ZnONP-90	5.8 ± 0.28	7.8 ± 1.2
PA-O_2_-ZnONP-60	7 ± 0.86	6 ± 0.5
PA-O_2_-ZnONP-90	7.6 ± 1.4	6.83 ± 1.1

The UV protection capability of the uncoated and coated PA was evaluated based on AATCC Test Method 183:2010^20^. Hence, the UPF for uncoated PA was found to be 12.5, displaying high transmittance in both UVB and UVA (Table 5). However, upon the coating of PA (without plasma treatment) with coating layer composed of ZnONP-TPP-TBHP ( PA- ZnONP sample) achieved UPF value of 27 displaying good blocking of UV transmittance in the both regions UVA and UVB compared to the uncoated PA, recording more than two-fold enhancement for the protection against harmful UV rays. This UV protection ability could be attributed to the intrinsic ZnONPs feature for absorbing UV rays^14^. This is in addition to the role of compounds existed in molokhia stems extract for UV shielding^14^. Interestingly, after functionalization of PA surface via plasma treatment, the UV shielding properties was strongly enhanced achieved good UV rays shielding (Table 5). Thus, the UPF values increased recording higher values of 39.6 and 32.6 for PA-N_2_-ZnONP-60 and PA-O_2_-ZnONP-90, respectively. This higher UV shielding properties were ascribed to the role for plasma treatment for increasing nanocomposite-based coating layer add on % and adhesion on PA surface which is responsible for UV protection. Interestingly, the developed nanocomposite-based coating for PA-N_2_-ZnONP-60 sample achieved good UV shielding properties recording UPF value of 39.6 which is comparable to other reported coatings system for different fabrics such as AgNPs coated cotton fabric (UPF value of 6.7)^24^, Curcumin/TiO_2_ nanocomposite-modified cotton fabric with GPTMS (UPF value of 38)^25^, Eu-MOF@Q-Cotton (UPF value of 37.8)^20^, VRF (UPF value of 50)^26^, cotton treated with TiO_2_/Au1% (UPF value of 45.58)^27^, CCF/GO-PVA (UPF value of 40.2)^28^, and cotton treated NanoZnO-3 (UPF value of 30)^29^. This reveals that the plasma treatment was effective tool for incorporation of effective fire safety, antibacterial and UV protection for synthetic PA fabrics.

**Table 5:** The UV protection properties for PA and their coated fabrics according to AATCC Test Method 183:2010.

Sample Code	UVA Transmittance (%)	UVB Transmittance (%)	UPF
PA	10.3	6.6	12.5
PA- ZnONP	4.5	3.2	27
PA-N_2_-ZnONP-60	3.2	2.2	39.6
PA-N_2_-ZnONP-90	4.7	3.3	26.8
PA-O_2_-ZnONP-60	4.8	3.8	24
PA-O_2_-ZnONP-90	3.5	2.8	32.6

Interestingly, to evaluate the practical application value of the coated PA fabric, the durability experiment was conducted via washing of the PA-O_2_-ZnONP-90 for five consecutive washing cycles based on our reported method^14^. Then, upon each washing cycle, the LOI test as flame retardant test was conducted and data was listed in Table S2. It is obvious from the results, the good durability of coated PA after cycle 2, recording LOI value of 14%. However, after cycle three LOI value was strongly decreased. Therefore, the coated PA could be considered as semi- durable and further enhancement is required to improve the adhesion between coating layer and PA to overcome this limitation.

## Conclusion

Gas plasma was used for surface activation of synthetic polyacrylic fabric to afford effective incorporation of new smart functions for the fabric. Two different gas plasma; O_2_ and N_2_ were implemented for treatment and time exposure was varied. The influence of plasma gas type and time exposure was investigated for coating layer add on %. Coating layer composite composed from greenly prepared ZnONPs, sodium tripolyphosphate and tetra-n-butylammonium hexafluorophosphate was facilely developed. The fresh plasma treated fabrics were directly coated with coating layer for incorporation of fire safety, antibacterial and UV proportion features for polyacrylic fabric. The coated fabrics achieved good flame retardancy behavior recording significant decrease in burning rate by 83%. The flame retardancy action was attributed to the synergistic effect between phosphate-based compounds, which triggers the polyacrylic fabrics for compact and coherent char layer formation. Moreover, record good reduction in the emission of toxic gases. The capability of coated PA for inhibition of bacterial growth was significantly increased due to coating layer components recording clear antibacterial inhibition zone of 7.6 and 7.8 mm for *Staphylococcus aureus* and *Escherichia coli* bacteria, respectively, compared to blank polyacrylic sample. Interestingly, the coating layer affords good shielding against UV rays, achieving improvement in UPF value of 39.6 compared to 12.5 for uncoated PA fabric. The developed coating layer with aid of plasma treatment reinforced the polyacrylic fabrics strength.

## Supplementary Information

Below is the link to the electronic supplementary material.

Supplementary Material 1

## Data Availability

All data generated or analyzed during this study are included in this published article and supporting information .
