# Supplementary material for: Plasma tool as green route for incorporation of flame retardancy and ultraviolet protection of textile fabrics
Source: Sci Rep. 2026 Apr 16;16:12474. doi: 10.1038/s41598-026-47539-x (PMC13087197; doi:10.1038/s41598-026-47539-x)
Supplement: Supplementary file 1 — Supplementary Material 1 [file 41598_2026_47539_MOESM1_ESM.docx]

**Supporting information**

**Plasma Tool as Green Route for Incorporation of Flame Retardancy and Ultraviolet Protection of Textile Fabrics**

Ahmed M. Abdel-Razik,^1^ Hanaa E. Nasr,^2^ Nour F. Attia*^3^

*^1^Materials Testing and Surface Chemical Analysis Laboratory, Chemistry Division, National Institute for Standards, 136, Giza 12211, Egypt*

*^2^Department of Polymers and Pigments, National Research Centre, Dokki,* Giza, 12622, Egypt

*^3^Gas Analysis and Fire Safety Laboratory, Chemistry Division, National Institute for Standards, 136, Giza 12211, Egypt*

*Corresponding Author

Prof. Dr. Nour F. Attia

Email Address: drnour2005@yahoo.com


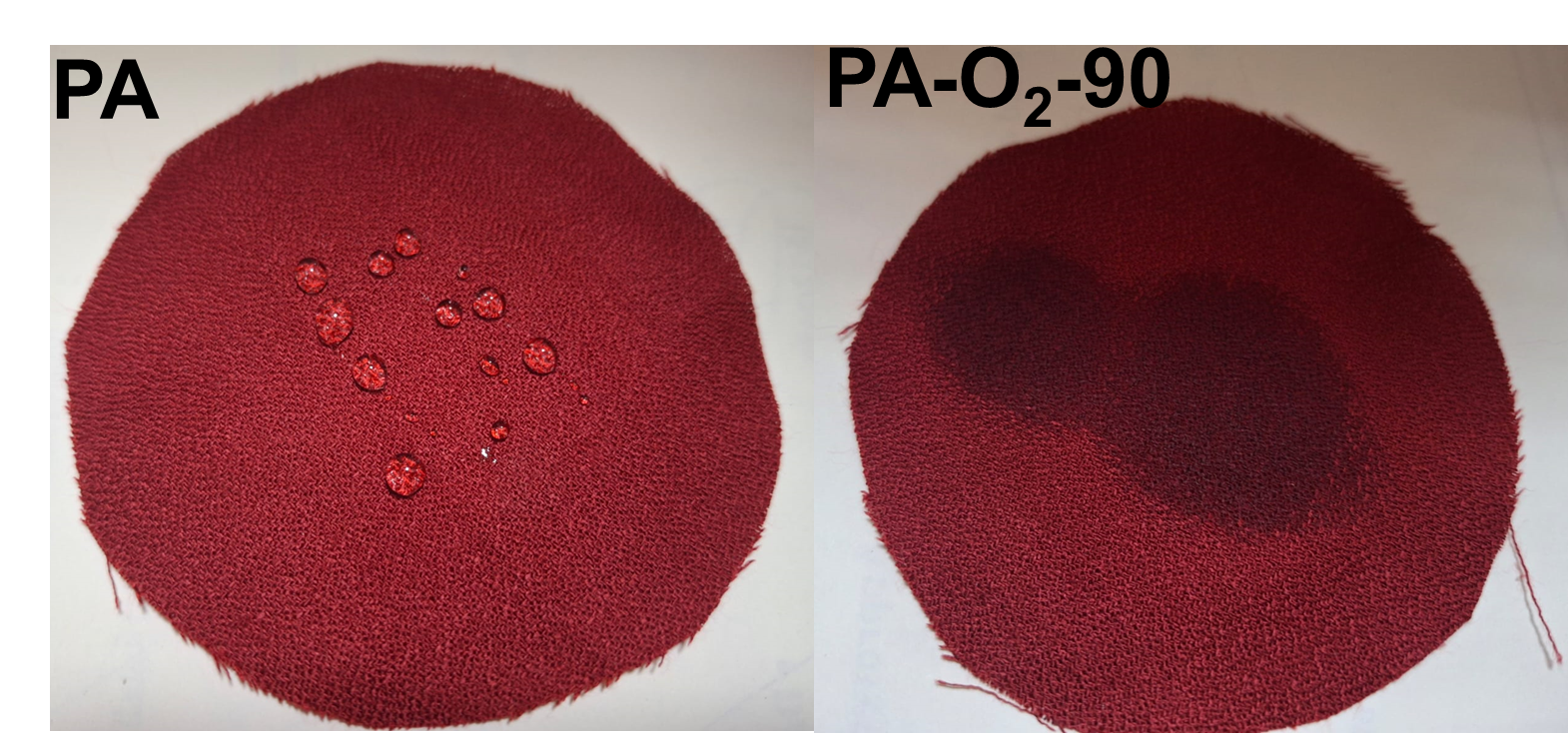


**Fig.S1.** Digital photos displaying the hydrophobicity of plasma untreated PA and the wettability of O_2_ plasma treated PA (PA-O_2_-90).

| Sample Code | CO (ppm) | Standard Deviation | | CO_2_ (%) | Standard  Deviation | | NOx (ppm) | | Standard  Deviation | | | NO (ppm) | | Standard  Deviation | | | SO_2_(ppm) | | | Standard  Deviation | | |
| --- | --- | --- | --- | --- | --- | --- | --- | --- | --- | --- | --- | --- | --- | --- | --- | --- | --- | --- | --- | --- | --- | --- |
| PA  PA- ZnONP  PA-N_2_-ZnONP-60 | 810  781.3  780.3 | 3.46  0.57  1.15 | 0.98  0.83  0.85 | | 0.0057  0.0057  0.011 | 13  9.6  9 | | 0.17  0.54  0.26 | | | 19.7  12  11.7 | | 0.57  0.1  1.15 | | | 91  75  78 | | | 1.73  2  1 | | |  |
| PA-N_2_-ZnONP-90 | 820.3 | 1.15 | 0.92 | | 0.011 | 12 | | 0.55 | | | 16.7 | | 0.57 | | | 80.3 | | | 0.57 | | |  |
| PA-O_2_-ZnONP-60 | 778.3 | 1.154 | 0.81 | | 0.0057 | 9.3 | | 0.57 | | | 13 | | | | 0.17 | 77.6 | | 0.57 | | |  |  |
| PA-O_2_-ZnONP-90 | 775 | 1.73 | 0.82 | | 0.0057 | 7.6 | | | | 0.57 | 11.7 | | | | 0.057 | 75.6 | | 0.57 | | |  |  |

**Table S1.** Toxic gases emission during LOI test.


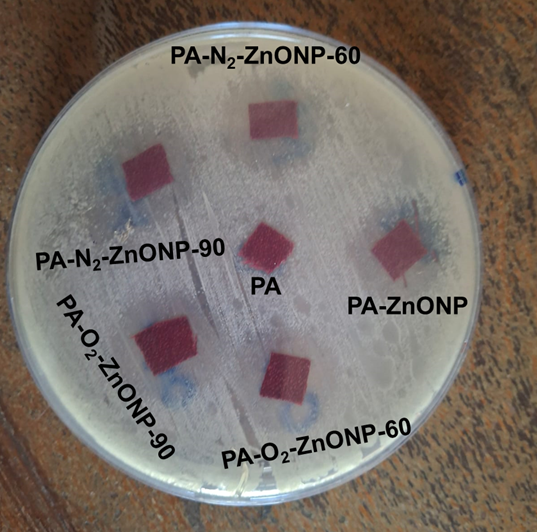


**Fig. S2.** The digital photos of the clear antibacterial inhibition zone of PA fabric and coated PA fabric against Escherichia coli bacteria.

**Table S2.** Displaying washing cycles of PA-O_2_-ZnONP-90 and corresponding LOI data after each cycle

| Sample Code | No. of washing cycle | No. of washing cycle | No. of washing cycle | No. of washing cycle | No. of washing cycle | No. of washing cycle |
| --- | --- | --- | --- | --- | --- | --- |
| PA-O_2_-ZnONP-90 | 0  WC | 1  WC | 2  WC | 3  WC | 4  WC | 5  WC |
| LOI (%) | 16.5 | 14 | 14 | 13.5 | 13.5 | 13.5 |
